# Supplementary material for: Cloud BioLinux: pre-configured and on-demand bioinformatics computing for the genomics community
Source: BMC Bioinformatics. 2012 Mar 19;13:42. doi: 10.1186/1471-2105-13-42 (PMC3372431; doi:10.1186/1471-2105-13-42)
Supplement: Additional file 1 — Supplementary 1 Cloud BioLinux software documentation in the form of a mini, self-contained website. Users need to download and uncompress the .zip file, and open through a web browser the "index.html" file available on the main directory. (ZIP 1823 kb). [file 1471-2105-13-42-S1.ZIP › Cloud-BioLinux-Package-Documentation/docs/check.html]

Bio-Linux Software Documentation Pages

Back to search form

## check

|  |  |
| --- | --- |
| Name | check |
| Description | **check** is a part of the Glimmer package, for finding genes in microbial DNA, especially the genomes of bacteria, archaea, and viruses.  This program checks for in-frame stop codons  **References:**  Salzberg SL, Delcher AL, Kasif S, White O: Microbial gene identification using interpolated Markov models. Nucleic Acids Res. 1998 Jan 15;26(2):544-8. [Entrez]    Delcher AL, Harmon D, Kasif S, White O, Salzberg SL: Improved microbial gene identification with GLIMMER. Nucleic Acids Res. 1999 Dec 1;27(23):4636-41. [Entrez] |
| Homepage | http://www.tigr.org/software/glimmer/ |
| Remote Documentation | http://www.tigr.org/software/glimmer/glimmer.readme |
